# FgRab5 and FgRab7 are essential for endosomes biogenesis and non-redundantly recruit the retromer complex to the endosomes in *Fusarium graminearum*

**DOI:** 10.1007/s44154-021-00020-3

**Published:** 2021-12-06

**Authors:** Yakubu Saddeeq Abubakar, Han Qiu, Wenqin Fang, Huawei Zheng, Guodong Lu, Jie Zhou, Zonghua Wang, Wenhui Zheng

**Affiliations:** 1 State Key Laboratory of Ecological Pest Control for Fujian and Taiwan Crops, Fujian Agriculture and Forestry University, Fuzhou, China; 2 College of Life Sciences, Fujian Agriculture and Forestry University, Fuzhou, China; 3 Department of Biochemistry, Faculty of Life Sciences, Ahmadu Bello University, Zaria, Nigeria; 4 Institute of Oceanography, Minjiang University, Fuzhou, China; 5 Key Laboratory of Integrated Pest Management for Fujian-Taiwan Crops, Ministry of Agriculture, College of Plant Protection, Fujian Agriculture and Forestry University, Fuzhou, China

**Keywords:** *Fusarium graminearum*, Rab GTPases, Retromer, vesicle trafficking

## Abstract

The retromer complex, composed of the cargo-selective complex (CSC) Vps35-Vps29-Vps26 in complex with the sorting nexin dimer Vps5-Vps17, mediates the sorting and retrograde transport of cargo proteins from the endosomes to the trans-Golgi network in eukaryotic cells. Rab proteins belong to the Ras superfamily of small GTPases and regulate many trafficking events including vesicle formation, budding, transport, tethering, docking and fusion with target membranes. Herein, we investigated the potential functional relationship between the retromer complex and the 11 Rab proteins that exist in *Fusarium graminearum* using genetic and high-resolution laser confocal microscopic approaches. We found that only FgRab5 (FgRab5A and FgRab5B) and FgRab7 associate with the retromer complex. Both FgVps35-GFP and FgVps17-GFP are mis-localized and appear diffused in the cytoplasm of *ΔFgrab5A*, *ΔFgrab5B* and *ΔFgrab7* mutants as compared to their punctate localization within the endosomes of the wild-type. FgRab7 and FgRab5B were found to co-localize with the retromer on endosomal membranes. Most strikingly, we found that these three Rab GTPases are indispensable for endosome biogenesis as both early and late endosomes could not be detected in the cells of the mutants after FM4-64 staining of the cells, while they were very clearly seen in the wild-type PH-1. Furthermore, FgRab7 was found to recruit FgVps35 but not FgVps17 to the endosomal membranes, whereas FgRab5B recruits both FgVps35 and FgVps17 to the membranes. Thus, we conclude that the Rab proteins FgRab5A, FgRab5B and FgRab7 play critical roles in the biogenesis of endosomes and in regulating retromer-mediated trafficking in *F. graminearum*.

**Supplementary Information:**

The online version contains supplementary material available at 10.1007/s44154-021-00020-3.

## Introduction

One of the major threats to the quality and yield of wheat globally is Fusarium Head Blight (FHB) caused by the fungal pathogen *Fusarium graminearum*. In addition to wheat, *F. graminearum* also attacks and causes massive destruction of other small grain cereals such as barley (Yang et al. 2013). The grains of the infected cereals are usually unsafe for consumption as the pathogen releases life-threatening mycotoxins such as zearalenone and deoxynivalenol (DON) that contaminate the grains (Alisaac et al. 2020). Today, FHB stands as a global concern, causing great economic losses and posing threats to global food security (Chakraborty and Newton 2011), hence the urgent need to tackle this problem. Understanding the biology and molecular mechanisms regulating the pathogenesis of *F. graminearum* will help to uncover different drug targets that could be promising in the control and management of FHB.

Vesicle trafficking is an essential process that mediates a lot of cell functions (Ma and Burd 2020). However, this process changes the positions of membrane proteins to the target organelles. Certain transport machineries, such as the retromer complex, are therefore required to convey these proteins back to their appropriate locations to exert their biological roles (Abubakar et al. 2017). The retromer protein complex mediates the vesicular transport of some membrane proteins (cargoes) from the endosomes to the trans-Golgi network (TGN) and the plasma membrane (Liu 2016). It is a heteropentamer built from two major subcomplexes. The core subcomplex is a trimer consisting vacuolar protein sorting 35 (Vps35), Vps29 and Vps26, often referred to as the cargo-selective complex (CSC); while the other component is a dimer of sorting nexin (SNX) proteins (such as Vps5 and Vps17) that binds to phosphatidylinositol 3-phosphate-enriched endosomal membranes during cargo sorting (van Weering et al. 2012; Shin et al. 2017). A lot of evidences indicate that cargo selectivity depends on defined combination of the CSC with the SNX family members in a given vesicle transport pathway (Abubakar et al. 2017). Deletion of any one of the genes coding for the retromer subunits significantly perturbs the growth, sexual and asexual developments and pathogenesis of *F*. *graminearum* (Zheng et al. 2016).

Rab GTPases constitute the largest subfamily of Ras-related small GTPases that are evolutionarily conserved and have been implicated in various cellular functions including growth, protein trafficking, transmembrane signal transduction, targeting and fusion of vesicles on membrane-bound organelles (Stenmark 2009; Pfeffer 2017). Physiologically, Rab proteins exist as either activated (GTP-bound) or inactivated (GDP-bound) form within the intracellular environment, and the activation/inactivation cycle is controlled by upstream regulators (Bhuin and Roy 2014). Usually, the inactive Rab GTPases are phosphorylated (activation) by GEFs (Guanine-nucleotide Exchange Factors) while the active forms are dephosphorylated (inactivation) by GAPs (GTPase Activating Proteins) (Bhuin and Roy 2014; Wandinger-Ness and Zerial 2014). Rab proteins localize to different membrane-bound subcellular compartments and some of them are considered as markers for such organelles (Pfeffer 2017). Members of this protein family are essential for vesicle trafficking during endocytosis/exocytosis as they are virtually involved in coordinating all stages of these cellular events, ranging from cargo sequestration, through vesicle budding, uncoating, mobility and fusion at the target membrane (Stenmark 2009). Furthermore, Rab GTPases play important physiological roles and determine the pathogenicity of many phytopathogens. In *F. graminearum*, for example, six *RAB* mutants including *ΔFgRab5A*, *ΔFgRab5B*, *ΔFgRab6*, *ΔFgRab7*, *ΔFgRab8* and *ΔFgRab11* have drastically reduced mycelial growth and morphology, sexual and asexual developments, and lost their pathogenicity compared to the wild type (PH-1) (Zheng et al. 2015). Mutants of *RAB2* gene have inhibited vesicular transport from the endoplasmic reticulum to the Golgi complex (Tisdale et al. 1992). Rab4 controls the fast recycling from the early endosomes (EEs) or recycling endosomes (REs) directly back to the plasma membrane (Van Der Sluijs et al. 1991; van der Sluijs et al. 1992). FgYptA is mainly localized in the endosomes while FgRabX has cytosolic/granular localization and exhibits a diffuse pattern in the cytoplasm. Rab5 and Rab7 are the most important organelle markers within the endocytic pathway where the former serves as the marker for EEs while the latter is for late endosomes (LEs) (Rink et al. 2005; Poteryaev et al. 2010). Golgi transport is largely dependent on the action of Rab6 (Short et al. 2002). Rab8 controls the delivery of basolateral secretory traffic from the TGN to the REs along the TGN-RE-plasma membrane route, and is localized preferentially at the hyphal tips and conidia in *F. graminearum*, suggesting a role in polarized growth (Henry and Sheff 2008). We previously demonstrated in the rice blast fungus *Magnaporthe oryzae* that the two isoforms of MoRab5 (MoRab5A and MoRab5B, otherwise known as MoRab51 and MoRab52 respectively) are indispensable for vesicle trafficking, growth, asexual reproduction and pathogenicity of the fungus, and the proteins were found to be functionally non-redundant (Yang et al. 2017).

It is therefore clear that both Rab GTPases and the retromer complex mediate vesicle transport in different organisms. This suggests that Rab GTPases have close functional relationship with the retromer complex. In line with this observation, a previous study in *M. oryzae* demonstrates that the retromer complex is recruited to the vacuolar membrane by the Rab GTPase MoYpt7, and the two co-localize on vacuolar and late endosomal membranes (Wu et al. 2021). Similarly, the yeast Ypt7 protein is linked to retromer-mediated receptor recycling and fusion at the LE (Balderhaar et al. 2010). In mammals, the retromer interacts with many other proteins and it is functionally and mechanistically diverse in mediating distinct membrane trafficking pathways in a variety of cellular processes (Harbour and Seaman 2011; Liu 2016). Rab7-GTP binds to the CSC; and interfering with Rab7 function causes dissociation of the CSC but not the SNX dimer from membranes; however, Rab5-GTP does not interact with the CSC but perturbation of Rab5 function causes dissociation of both the SNX and CSC subcomplexes from membranes, through inhibition of a pathway involving phosphatidylinositol 3-kinase (Rojas et al. 2008). Arabidopsis has three *VPS35* homologue genes, *VPS35A*, *VPS35B* and *VPS35C* (Jaillais et al. 2007). The Vps35A protein interacts with Arabidopsis Rab7 only in its active (GTP-bound) form on endosomal membranes (Zelazny et al. 2013). Therefore, it can be understood from the different published data that the retromer complex requires different Rab proteins in different organisms to exert different functions. The present study puts forward a comprehensive investigation of the direct relationship between the retromer complex and the various Rab GTPases in *F. graminearum*. Of the 11 Rab GTPases present in the fungus (Zheng et al. 2015), we found that only FgRab7 and the two isoforms of FgRab5 (FgRab5A and FgRab5B, otherwise known as FgRab51 and FgRab52, respectively), mediate retromer localization and stability. They are critically required not only for endosomes biogenesis, but also for recruitment of the retromer complex to the endosomal membranes.

## Results

### Retromer complex does not require FgRab2, FgRab4, FgRab8, FgRabX and FgYptA for its normal intracellular localization

**Fig. 1:**
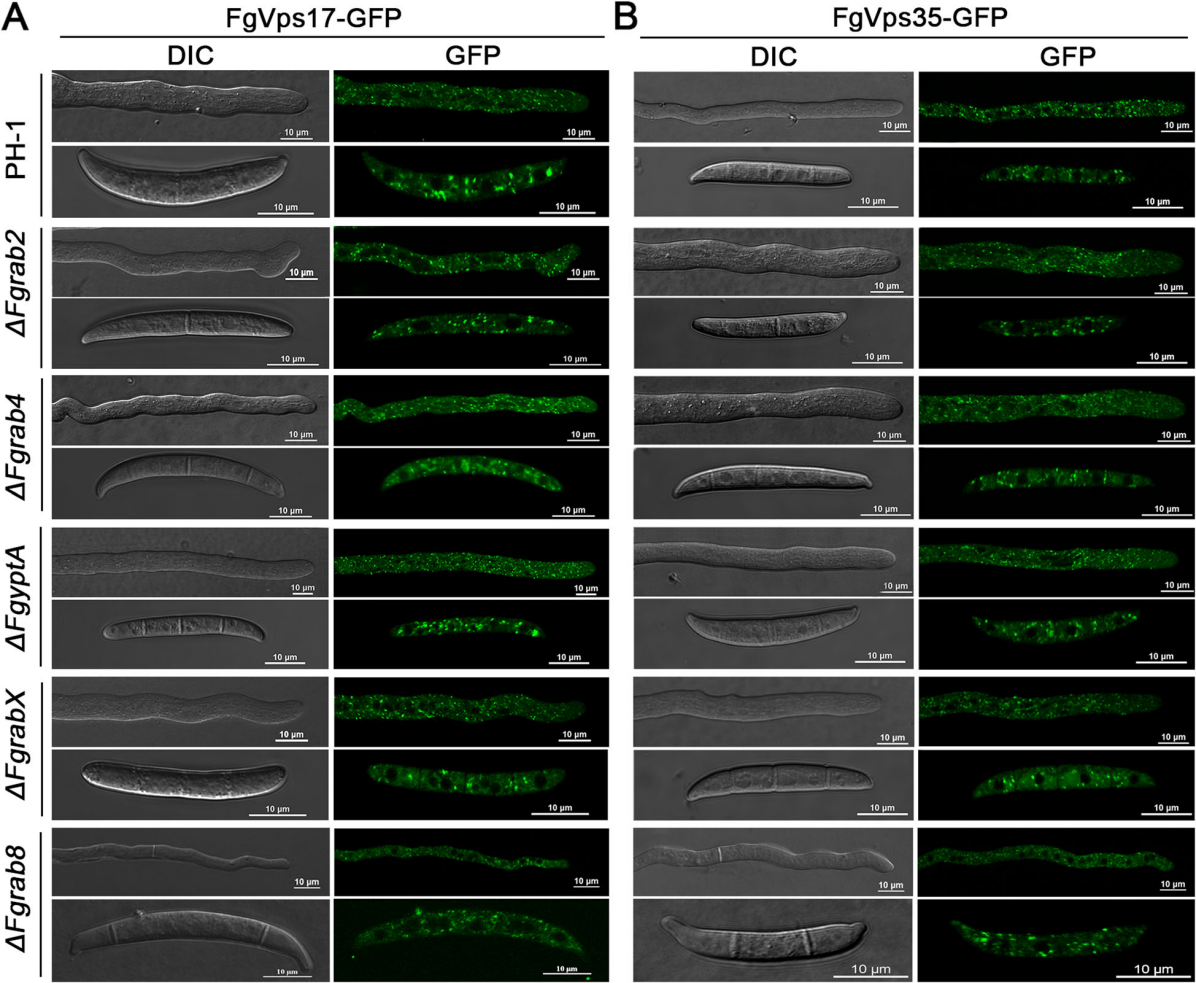
The Rab GTPases FgRab2, FgRab4, FgYptA, FgRabX and FgRab8 are not required for the punctate localization of the retromer complex in *F. graminearum*. (A) Subcellular localization of FgVps17-GFP in the growing hyphae and conidia of PH-1 (wild-type), *ΔFgrab2*, *ΔFgrab4*, *ΔFgyptA*, *ΔFgrabX* and *ΔFgrab8* mutants. (B) Subcellular localization of FgVps35-GFP in the growing hyphae and conidia of PH-1 (wild-type), *ΔFgrab2*, *ΔFgrab4*, *ΔFgyptA*, *ΔFgrabX* and *ΔFgrab8* mutants.

A previous study from our laboratory established that only 11 Rab GTPase genes exist in *F. graminearum* (including *FgRAB1, FgRAB2, FgRAB4, FgRAB5A* (*FgRAB51*)*, FgRAB5B* (FgRAB52)*, FgRAB6, FgRAB7, FgRAB8, FgRAB11, FgRABX and FgYPTA*) (Zheng et al. 2015). To investigate how the products of these genes relate with the retromer complex, we checked the subcellular localization of the retromer complex in the deletion mutants of the Rab genes, respectively, with the exception of *FgRAB1* and *FgRAB11* (whose deletion mutants could not be generated) and *Fgrab6* mutant (which grows so slowly that it is difficult to obtain adequate mycelia for protoplast preparation) (Zheng et al. 2015). Specifically, we visualized the localizations of FgVps35-GFP (the central subunit of the retromer CSC) and FgVps17-GFP (a SNX protein associated with the retromer complex) in the various *rab* mutants used. Our results demonstrate that FgVps35-GFP and FgVps17-GFP localize as punctate structures in the hyphae and conidia of the PH-1 (Fig. 1). The localizations of these retromer subunits remain unchanged in the absence of FgRab2, FgRab4, FgRab8, FgRabX and FgYptA proteins (Fig. 1 A and B), suggesting that these Rab proteins are dispensable for normal localization of the retromer complex in *F. graminearum*. It also suggests with high likelihood that the biological functions of the retromer complex are independent of these Rab GTPases.

### FgRab5A, FgRab5B and FgRab7 are essential for the punctate localization of the retromer complex

**Fig. 2:**
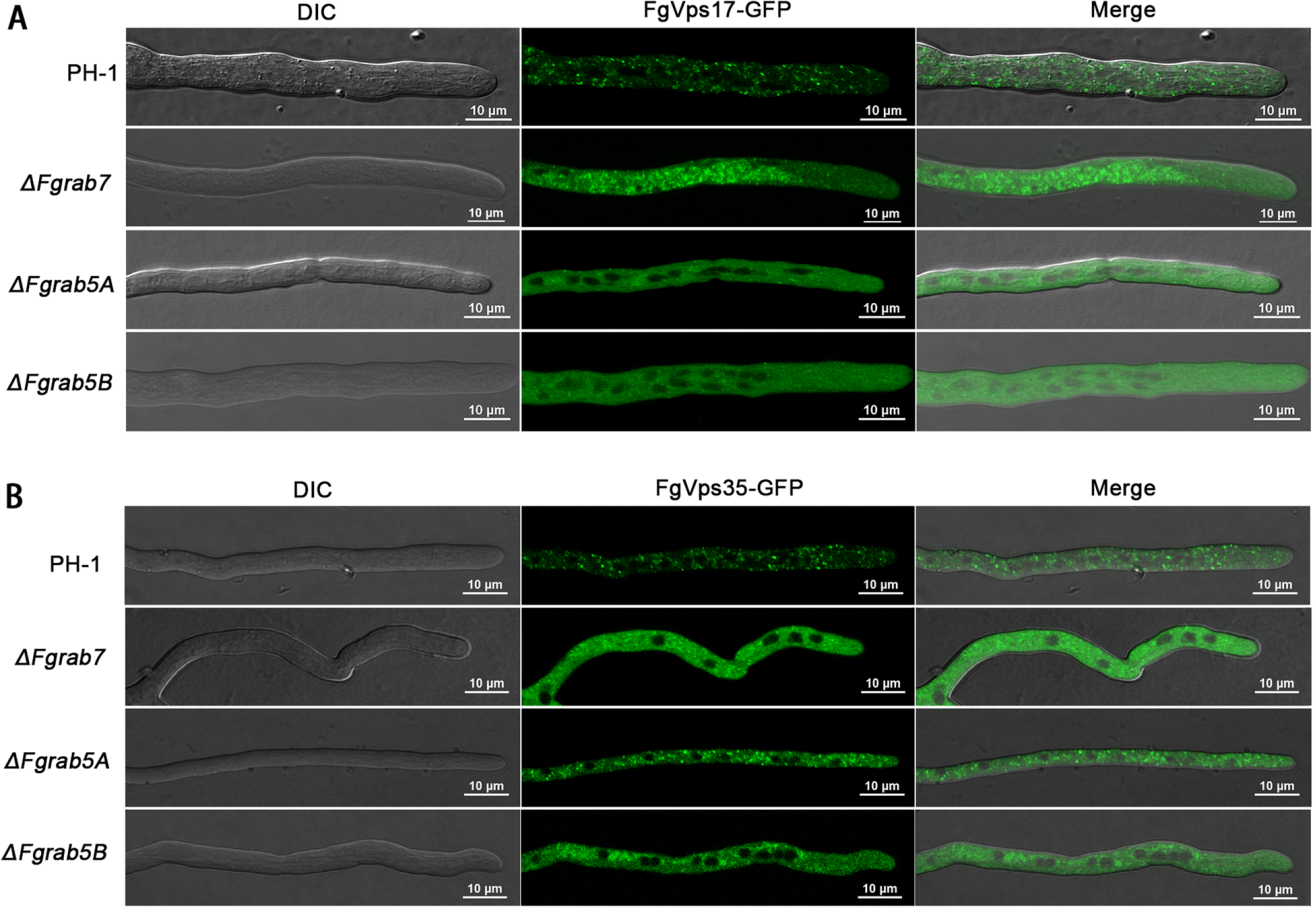
The Rab GTPases FgRab7, FgRab5A and FgRab5B are important for the punctate localization of the retromer complex in *F. graminearum*. (A) Subcellular localization of FgVps17-GFP in the growing hyphae of PH-1 (wild-type), *ΔFgrab7*, *ΔFgrab5A* and *ΔFgrab5B* mutants. (B) Subcellular localization of FgVps35-GFP in the growing hyphae of PH-1 (wild-type), *ΔFgrab7*, *ΔFgrab5A* and *ΔFgrab5B* mutants.

Similar to the previous approach, we checked the intracellular location of the retromer complex (FgVps35-GFP and FgVps17-GFP) in the deletion mutants of *FgRAB5A, FgRAB5B* and *FgRAB7* in relation to its localization in the wild-type. Unlike what was observed in the other five mutants presented, the FgVps35-GFP and FgVps17-GFP were found to be impaired or lose their normal punctate appearance due to deletions of *FgRAB5A, FgRAB5B* and *FgRAB7* genes (Fig. 2). Consistently, our time-lase video imaging of the hyphal cells further confirmed these results (Videos S1-8). The GFP-tagged subunits were rather observed to be ubiquitously expressed in the fungal hyphae, but undetected in the endosomes. The normal localization (endosomal localization (Zheng et al. 2016)) of these proteins was however observed in the control (PH-1) sample. This observation is consistently similar for both FgVps35-GFP and FgVps17-GFP (Fig. 2 A and B). This demonstrates that the retromer complex requires the Rab GTPases FgRab5A, FgRab5B and FgRab7 for its normal punctate localization in *F. graminearum*.

### Loss of retromer functions could be responsible for the impaired virulence of the Rab mutants

**Fig. 3:**
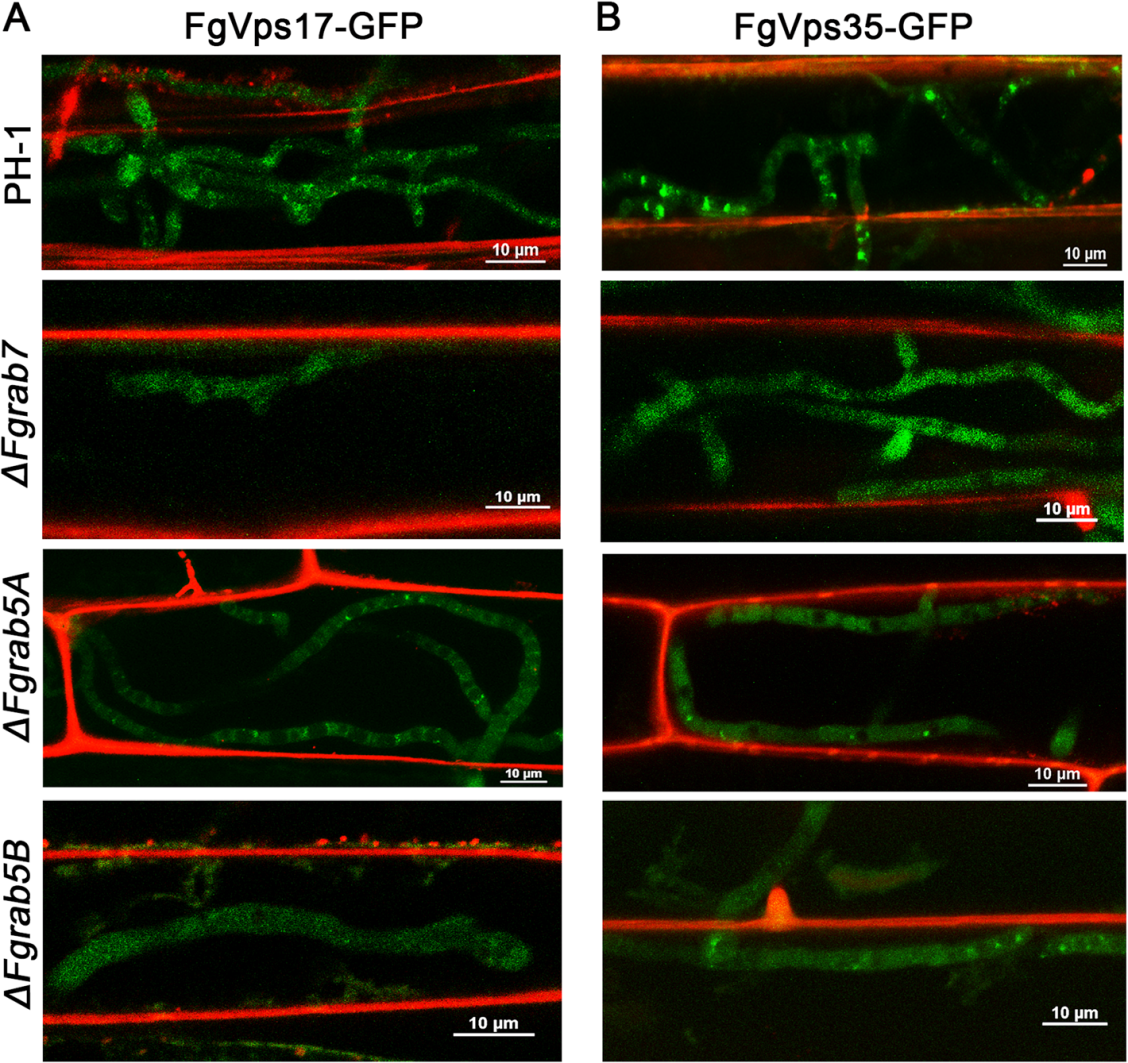
The Rab GTPases FgRab7, FgRab5A and FgRab5B are important for the punctate localization of the retromer complex during *F. graminearum* colonization in its host. Tissue cells of wheat coleoptiles were stained with PI (Propidium iodide) (red). (A) Subcellular localization of FgVps17-GFP in the invasive hyphae of PH-1 (wild-type), *ΔFgrab7*, *ΔFgrab5A* and *ΔFgrab5B* mutants. (B) Subcellular localization of FgVps35-GFP in the invasive hyphae of PH-1 (wild-type), *ΔFgrab7*, *ΔFgrab5A* and *ΔFgrab5B* mutants.

The results presented above simply suggest that retromer-mediated protein sorting is blocked in the absence of FgRab5 and FgRab7 in *F. graminearum* hyphae, and we suspected that even during host plant infection by the mutants, the localization of retromer should also be altered. To test this, we inoculated wheat coleoptiles with hyphae from the PH-1 as well as from the three mutants, all expressing FgVps35-GFP and FgVps17-GFP, and stained the inoculated wheat coleoptiles with propidium iodide (PI) to clearly mark the host plasma membrane after 12 hours of inoculation. Confocal microscopy of the infected host cells reveals that FgVps35-GFP and FgVps17-GFP were spot-shaped in the cytoplasm of the PH-1 hyphae (Fig. 3A, 3B). However, the dot-like localizations of FgVps35-GFP and FgVps17-GFP became diffused within the cytosol in Δ*Fgrab7* mutant, whereas the spotted signals of FgVps35-GFP and FgVps17-GFP were obviously reduced in Δ*Fgrab5A* and Δ*Fgrab5B* mutants (Fig. 3A, 3B). Our research team previously showed that *ΔFgrab5A*, *ΔFgrab5B* and *ΔFgrab7* mutants had significantly reduced disease indices, compared to PH-1, when inoculated on wheat spikelets (Zheng et al. 2015). This defect is very similar to the virulence defect of *ΔFgvps35* and *ΔFgvps17* mutants (Zheng et al. 2016). Put together, these results suggest that the loss-of-virulence observed in both retromer and the Rab GTPase mutants could be the result of abolished retromer functions in the mutants.

### Retromer co-localizes with FgRab5 and FgRab7 on endosomal membranes

**Fig. 4:**
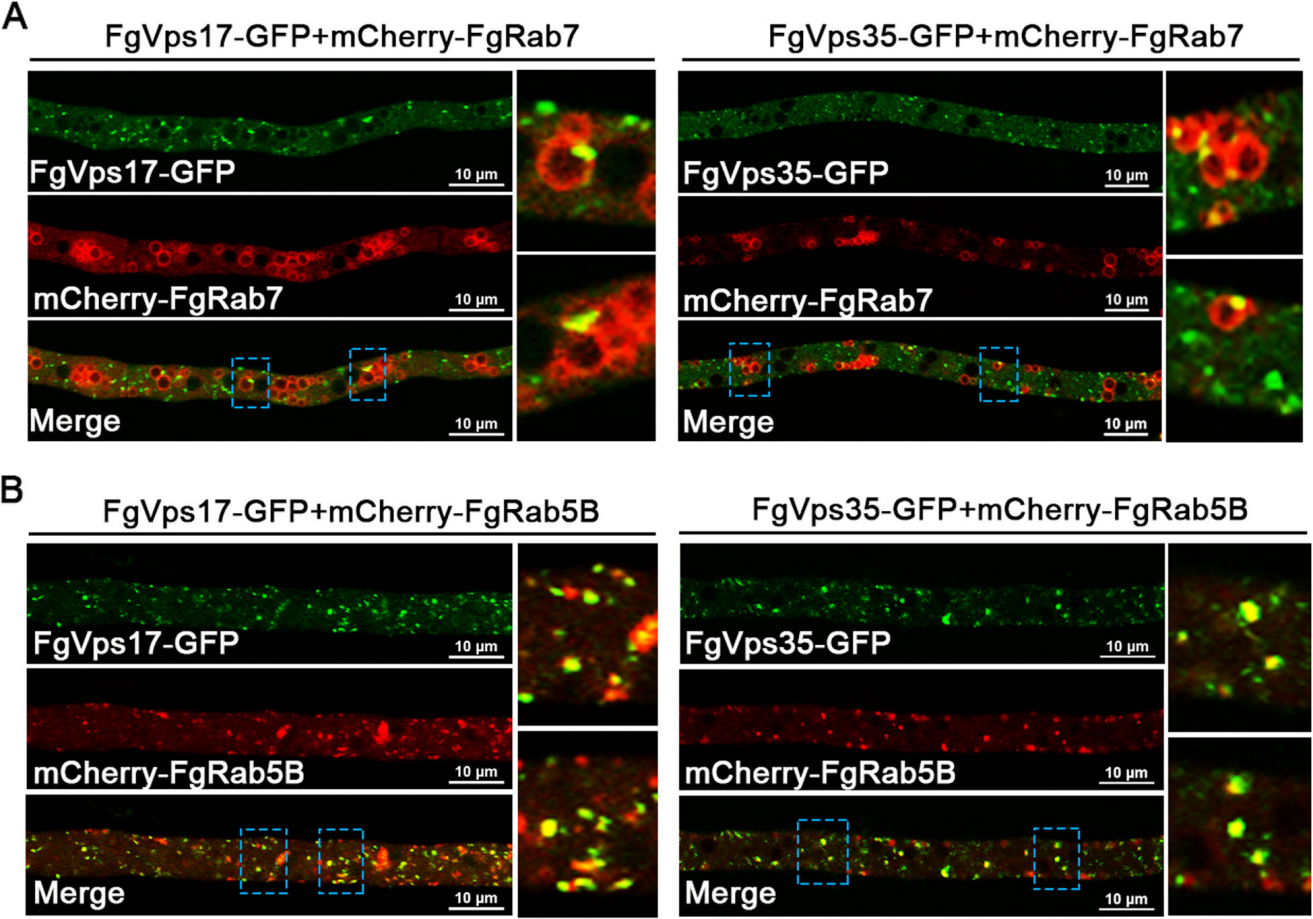
The Rab GTPases FgRab7 and FgRab5B colocalized with the retromer complex on late and early endosomes, respectively. (A) Punctate FgVps17-GFP and FgVps35-GFP fluorescent signals associate with mCherry-FgRab7-marked late endosomes (ring-like). Detailed views of each boxed region are shown by the right of the respective images. (B) Punctate FgVps17-GFP and FgVps35-GFP fluorescent signals co-localized with mCherry-FgRab5B-marked early endosomes (punctate). Detailed views of each boxed region are shown by the right of the respective images.

Our earlier findings here show the dependence of the retromer localization on FgRab5A, FgRab5B and FgRab7. Based on this, we expect positive co-localizations of these Rab GTPases with the retromer complex. To verify this, we tagged each of FgRab5B and FgRab7 with mCherry and generated mCherry-FgRab5B and mCherry-FgRab7 constructs, respectively. Each of these constructs was transformed into the FgVps35-GFP and FgVps17-GFP expressing strains for co-localization analysis. We found that mCherry-FgRab7 is localized on endosomal membrane (Fig. 4A) (generally, Rab5 and Rab7 are markers for early and late endosomes, respectively). This protein was observed to co-localize with both FgVps17-GFP and FgVps35-GFP. Similarly, mCherry-FgRab5B co-localized with the punctate FgVps17-GFP and FgVps35-GFP on the endosomal membrane (Fig. 4B). This is consistent with a previous study that shows that the retromer complex is localized to both early and late endosomes in *F. graminearum* (Zheng et al. 2016). Taken together, our data here signify that in *F. graminearum*, retromer co-localizes with FgRab5 and FgRab7 on early and late endosomal membranes, respectively.

### FgRab5A, FgRab5B and FgRab7 are required for endosomes biogenesis and retromer recruitment to the endosomal membranes

**Fig. 5:**
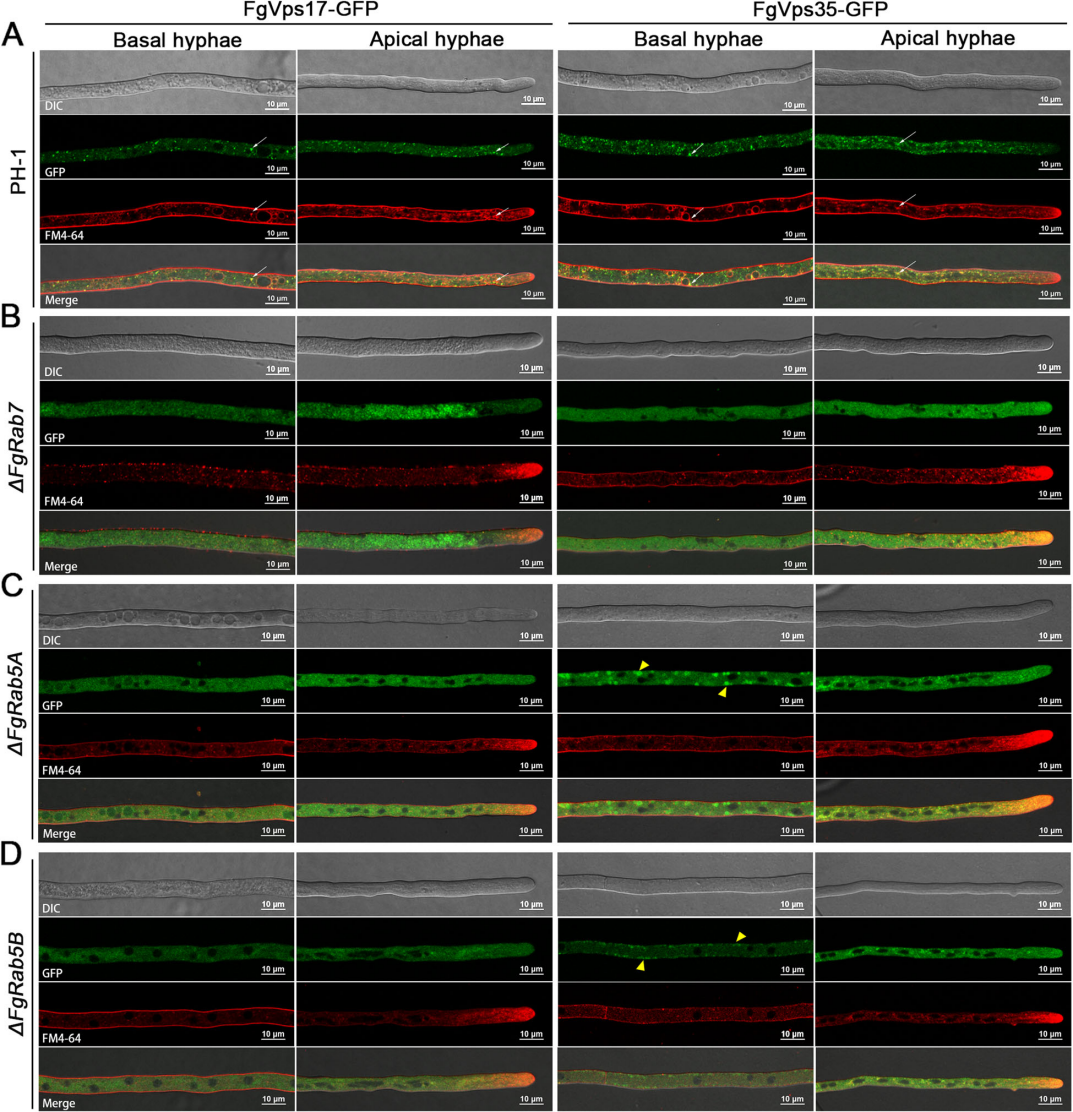
Deletion of *FgRAB7*, *FgRAB5A* or *FgRAB5B* blocks endosomes biogenesis and hence leads to mis-localization of the retromer complex to the cytoplasm. Endosomes of the *F. graminearum* hyphae were stained with FM4-64 (red). (A) In both basal and apical hyphae, FgVps17-GFP and FgVps35-GFP co-localized with the FM4-64-marked endosomes in the PH-1. White arrows indicate the co-localization sites. (B) In both basal and apical hyphae, FgVps17-GFP and FgVps35-GFP are distributed within the cytoplasm in the *ΔFgrab7* mutant. (C) In both basal and apical hyphae, FgVps17-GFP is distributed within the cytoplasm of the *ΔFgrab5A* mutant. Remarkably, in the *ΔFgrab5A* mutant, the FgVps35-GFP is distributed throughout the cytoplasm in apical hyphae while it is mis-localized to the plasma membrane (arrowheads) in the basal hyphae. (D) In both basal and apical hyphae, FgVps17-GFP is distributed in the cytoplasm of the *ΔFgrab5B* mutant. Remarkably, in the *ΔFgrab5B* mutant, the FgVps35-GFP is distributed in the cytoplasm of the apical hyphae, while it is mis-localized to the plasma membrane (arrowheads) in basal hyphae.

Retromer is localized to both EEs and LEs. Since the absence of FgRab5A, FgRab5B and FgRab7 mis-localizes the retromer complex to the cytoplasm, we hypothesized that this happened due to complete absence of the endosomes in the cells. To test this hypothesis, we used FM4-64, an amphiphilic styrene dye that stains plasma and endosomal membranes. In the PH-1, this dye clearly marked the endosomes, making them visible in both basal and apical hyphae under a confocal microscope (Fig. 5A). When merged with FgVps17-GFP and FgVps35-GFP, these endosomes co-localized with the retromer subunits in the PH-1 strain, further supporting that these circular structures are endosomes. In the *RAB* mutants, however, these structures are obviously undetectable after treatment of the hyphae with the dye (Fig. 5B, 5C and 5D). In *ΔFgrab5A* and *ΔFgrab5B* mutants, we detected the presence of FgVps35-GFP along the intracellular surface of the plasma membrane (Fig. 5C and 5D), suggesting that FgRab5 is required not only for the biogenesis of the early endosome but also for the recruitment of FgVps35 to the endosomal membrane.

**Fig. 6:**
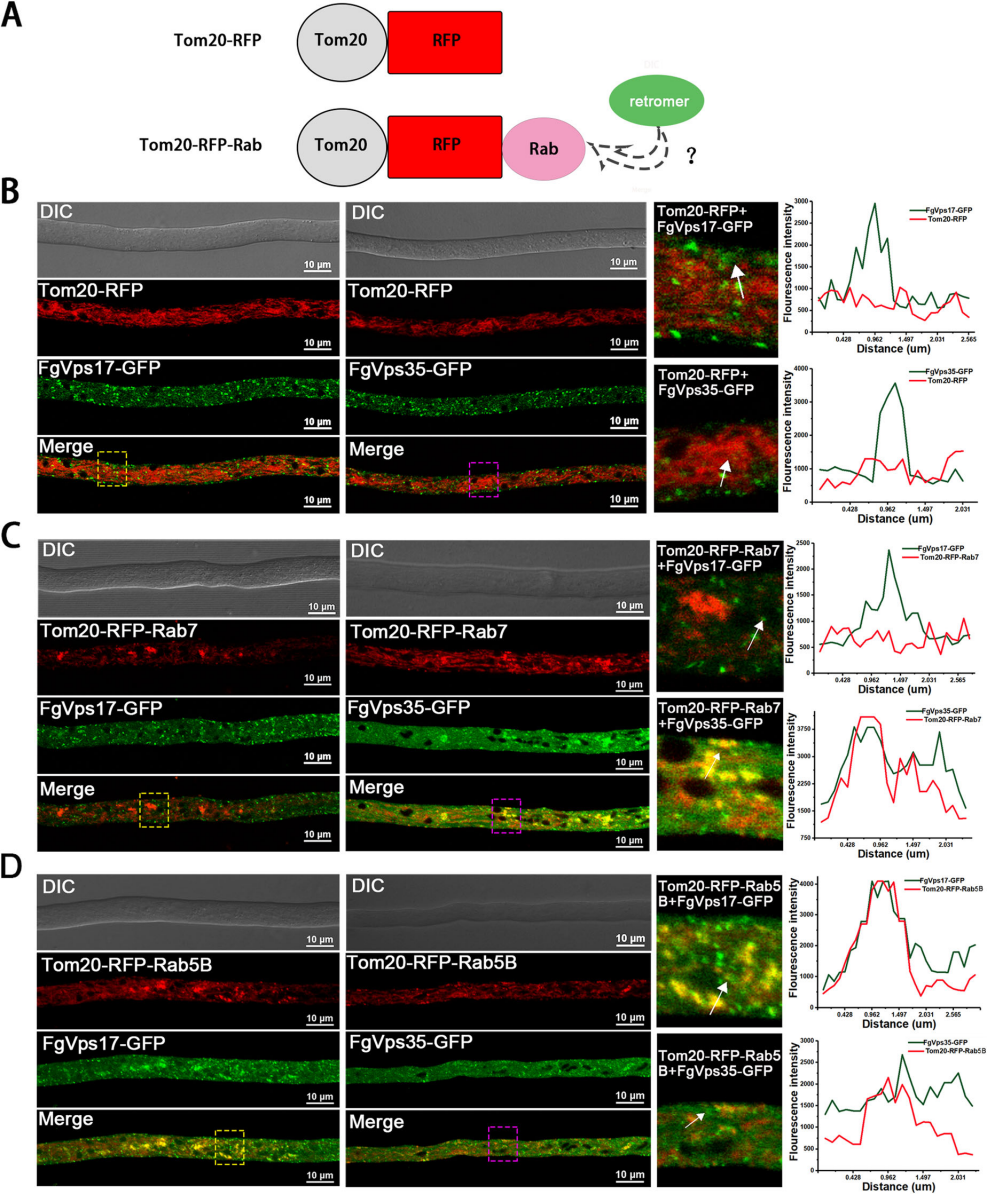
Further dissection of the roles of FgRab7 and FgRab5B in retromer recruitment to the endosomes. FgRab7 is required for the recruitment of FgVps35 but not FgVps17, whereas FgVps5B recruits both FgVps35 and FgVps17. (A) Schematic illustration of the ectopic targeting assay for Tom20-RFP and Tom20-RFP-Rab fusions. (B) Expressions of FgVps17-GFP and FgVps35-GFP in Tom20-RFP-expressing strains, respectively. Detailed views of each boxed region are shown by the right of the respective images. A line scan graph was generated at the indicated positions (arrow) to show the relative co-localizations of FgVps17-GFP and FgVps35-GFP with Tom20-RFP. (C) FgVps35-GFP is recruited to mitochondria in the hyphal cells expressing Tom20-RFP–tagged FgRab7 but not FgVps17-GFP. Detailed views of each boxed region are shown by the right of the respective images. A line scan graph was generated at the indicated positions (arrow) to show the relative co-localizations. (D) Both FgVps35-GFP and FgVps17-GFP are recruited to mitochondria in the hyphal cells expressing Tom20-RFP–tagged FgRab5B. Detailed views of each boxed region are shown by the right of the respective images. A line scan graph was generated at the indicated positions (arrow) to show the relative co-localizations.

To further verify the above results, we used a mitochondrial protein, Tom20, which binds to the outer mitochondrial membrane through its N-terminal transmembrane region, while its C-terminus is exposed to the cytoplasm (Abe et al. 2000; Yamamoto et al. 2011). We constructed Tom20-RFP and Tom20-RFP fused with FgRab5B (Tom20-RFP-Rab5B) and FgRab7 (Tom20-RFP-Rab7) (Fig. 6A) and co-transformed each of them with FgVps17-GFP and FgVps35-GFP into PH-1 respectively. As a control, Tom20-RFP does not alter the localization of the retromer (Fig. 6B). We found that FgRab5B and FgRab7 which were originally localized to endosomes are now anchored to the mitochondria through Tom20 (Fig. 6C-6D). However, when FgRab7 is anchored to the mitochondrial membrane by Tom20, FgVps35-GFP is observed to be partially recruited to the mitochondria by FgRab7 while the localization of FgVps17-GFP is not affected under this circumstance (Fig. 6C). Besides, when FgRab5B is anchored to the mitochondrial membrane, FgVps17-GFP and FgVps35-GFP are partially recruited to the mitochondrial membrane (Fig. 6D). Put together, these results indicate that the Rab GTPases FgRab5 and FgRab7 are indispensable for the biogenesis of endosomes and are involved in recruiting the retromer complex to the endosomal membranes in *F. graminearum*.

## Discussion

Retromer mediates retrograde transport of transmembrane protein cargos from early endosomes to the trans-Golgi network (TGN) and it is well conserved among different species (Seaman et al. 1998). Defects in retromer functions result in unnecessary degradation of some important receptors in the vacuole (Arighi et al. 2004). Rab GTPases are the key regulators of membrane trafficking and intracellular vesicular transport in eukaryotes (Hutagalung and Novick 2011). FgRab1, FgRab2 and FgRab6 are mainly localized to endoplasmic reticulum, Golgi and trans-Golgi, respectively, and they function in exocytosis pathways; FgRab8 and FgRab11 are localized at the hyphal tip of *F. graminearum*; while FgRab4, FgRab5A, FgRab5B, FgRab7, FgYptA can be found in early endosomes, plasma membranes and vacuoles and also regulate endocytosis (Zheng et al. 2015). In this study, we investigated the functional dependence of the retromer complex on the various Rab GTPases present in *F. graminearum*. We found that the normal localization of the retromer requires FgRab5A, FgRab5B and FgRab7 of the 11 Rab proteins that exist in the phytopathogen. As such, we further investigated the relationship between retromer and these three Rab proteins.

Like in the wild-type, the localizations of FgVps35-GFP and FgVps17-GFP proteins in each of Δ*Fgrab2*, Δ*Fgrab4*, Δ*Fgrab8*, Δ*Fgrabx* and Δ*FgyptA* mutants, are punctate in nature (Fig. 1A-1B). However, both FgVps35-GFP and FgVps17-GFP are mis-localized and FgVps17-GFP appeared more diffused than FgVps35-GFP in the cytoplasm of Δ*FgRab5A* and Δ*FgRab5B* mutants (Fig. 2A-2B). These florescent dots are highly accumulated in *ΔFgRab7* mutant hyphae (Fig. 2A-2B). Although FgRab5A and FgRab5B are homolog proteins, the retromer is mis-localized to the cytoplasm in the mutants of both proteins, indicating that the roles of these Rab proteins in ensuring the normal localization of the protein complex are not redundant. This observation agrees with previous findings in the rice blast fungus *Magnaporthe oryzae*, which demonstrated the non-redundant physiological and developmental functions of MoRab5A and MoRab5B in the fungus (Yang et al. 2017). Sill in *M. oryzae*, mis-localization of the retromer was similarly observed following deletion of *MoYPT7* (a homolog of *RAB7*) (Wu et al. 2021).

Even while inside the host tissues, the retromer complex was observed to be mis-localized and diffused in Δ*FgRab5A*, Δ*FgRab5B* and Δ*FgRab7* hyphae (Fig. 3A-3B). Independent studies demonstrated in *F. graminearum* that the pathogenicity of both the retromer and the Rab mutants were critically impaired and the impairments were quite similar (Zheng et al. 2015; Zheng et al. 2016). This means that the retromer mutants lack retromer function but possess functional FgRab5 and FgRab7, while the Rab mutants lack the functions of both the Rabs and the retromer complex. Therefore, the observed defects of the mutants are likely due to the absence of retromer functions, notwithstanding the fact that the Rab proteins could have other physiological functions as seen in the regulation of FgSnx41-FgSnx4 localization by FgRab5 (Zheng et al. 2018). Overall, these results indicate that the functions and punctate localization of the retromer complex are dependent on the Rab GTPases FgRab7, FgRab5A and FgRab5B in *F. gra*minearum.

In Arabidopsis, the Rab7 homolog RABG3f interacts with Vps35 to recruit the retromer CSC to the endosomal membrane (Zelazny et al. 2013). Also, the recruitment of retromer to endosomal membranes requires Rab7 in yeast and mammals (Liu et al. 2012; Modica et al. 2017). Retromer CSC binds to Rab7-GTP to get positioned on the endosome, while Rab5-GTP interacts with the 3-phosphatidylinositol kinase (PI3K)-related protein Vps34 rather than binding to CSC. Deletion of Rab5 inhibits the PI3K pathway causing dissociation of SNX and CSC with membrane (Rojas et al. 2008; Bean et al. 2015). Herein, we demonstrated that both FgVps35-GFP and FgVps17-GFP co-localized with mCherry-FgRab5B and mCherry-FgRab7 (Fig. 4A-4B). Most interestingly, we found that in the absence of any of the three Rab proteins, the endosomes could not be seen in the hyphae after staining with FM4-64, unlike their clear visibility in the PH-1, and this explains why the retromer complex is mis-localized to the cytoplasm after deletion of *FgRAB7*, *FgRAB5A* or *FgRAB5B* (Fig. 5A-5D). This simply suggests that both endosome biogenesis and the subsequent recruitment of the retromer complex to the endosomes require FgRab7 and FgRab5. In order to confirm this result, we constructed FgTom20-RFP vector and fused it with FgRab7 and FgRab5, and the two Rab proteins were observed to be anchored to mitochondria by FgTom20. If the Rab proteins really recruit retromer to the endosomes, retromer should, to some extent, be seen on the mitochondrial membranes. In agreement with this, our results showed that when FgRab5B was anchored to mitochondrial membrane, both FgVps17 and FgVps35 were detected on the mitochondria (Fig. 6D). We therefore speculate that when FgRab5B is anchored to the mitochondrial membranes, PI3K-related proteins get recruited to the mitochondrial membrane and PI3K promotes the generation of PI3P which is then recognized by the PX domain of FgVps17 and recruits this protein to the mitochondria. Finally, the SNX protein (FgVps17) then recruits the retromer CSC (FgVps35) to the mitochondrial membrane. However, when FgRab7 is anchored to the mitochondrial membrane, only FgVps35 is recruited to the mitochondrial membrane, and the localization of FgVps17 remains unchanged (Fig. 6C), which is consistent with the requirement of Rab7 for the recruitment of retromer CSC to the endosomes in yeast and mammals. This can also be explained from another perspective. Mon1-Ccz1 complex functions as a GEF for Rab7 and is recruited by Rab5-GTP to endosomes (Poteryaev et al. 2010; Langemeyer et al. 2018). Therefore, Rab5 recruits both Vps17 and Mon1-Ccz1 complex. The later then phosphorylates Rab7-GDP, activating it to Rab7-GTP which in turn recruits Vps35 to the endosomal membranes. Rabs will therefore be observed as recruiting Vps35.

## Materials and Methods

### Strains and culture conditions

In this study, the PH-1 isolate of *Fusarium graminearum* was used as the wild-type (WT) from which all other mutants were generated. All the *F. graminearum* strains used are listed in Table S1. The media used for culturing the various strains include complete medium (CM) and starch yeast medium (SYM) (Zheng et al. 2015). Conidia were harvested in liquid carboxymethyl cellulose (CMC) medium as reported in a previous study (Zheng et al. 2012).

### Protoplast preparation/Fungal transformation

The standard procedures for *F. graminearum* protoplast preparation and fungal transformation described by Hou and co-authors (Hou et al. 2002) were adopted.

### Targeted gene deletion

The *rab* gene deletion mutants used in this study were obtained from Dr. Huawei Zheng and the deletion strategy is clearly stated in his previous study (Zheng et al. 2015).

### GFP/mCherry fusion and gene complementation

The GFP/mCherry fusion constructs of FgRab and retromer proteins used in this study were obtained from Dr. Huawei Zheng and Dr. Wenhui Zheng, and the construction strategy was clearly explained in their previous study (Zheng et al. 2015).

### Live-cell imaging

For hyphal capture assays, a CM or SYM agar block containing the leading hyphae was excised from the appropriate fungal culture and placed upside down on a glass slide, with the fungal hyphae directly touching the surface of the slide and further incubated for 1-2 days. The hyphae on the glass slide, projecting from the block culture, were visualized under a confocal microscope.

For fluorescence assay of invasive hyphae, the PH-1 and the Rab mutant hyphal blocks were inoculated on SYM or CM agar and incubated for 4-6 days at 25°C. The mycelial blocks were then collected from the culture and further inoculated in liquid CMC medium and incubated at 25°C for 3 days with constant shaking at 150 rpm. The conidial suspension was filtered through a Miracloth. The harvested conidia were resuspended in sterile double-distilled water and adjusted to 10^7^ conidia/mL. A razor blade was used to cut the coleoptiles of the five-day-old wheat seedlings into thin slices. The slices were placed on carrier plates and 100 mL of the conidial suspension (or hyphal suspension for non-conidia-producing mutants) is added to each slice and the plates were incubated under humid condition for 12-36h at 25°C. The infected wheat slices were then stained with propidium iodide (PI) and visualized under a fluorescence microscope. FM4-64 (Molecular Probes, Eugene, OR, USA) was used for the staining of Spitzenkorper, plasma membrane, septum, EEs, LEs and vacuolar membrane, as well as for the examination of endocytosis, as described previously (Fischer-Parton et al. 2000).

### Time-lapse microscopy

Young growing hyphae and conidia were harvested from fresh cultures of the strains involved. These fungal parts were treated with 10μM FM4-64 and 100μM PI, and observed under a Nikon A1 laser confocal microscope (Tokyo, Japan) using time-lapse live-cell fluorescence imaging. The excitation/emission wavelengths used were 488 nm/500–550 nm for GFP and 561 nm/ 570–620 nm for mCherry, FM4-64 and PI. Sequence images were exported as avi files. Unless noted otherwise, all microscope images were taken within a single focal plane. To prevent cross-excitation, we performed scanning by sequentially firing the lasers for the channels to be used.

## Supplementary Information

**Additional file 1.**

**Additional file 2: Video S1.** The dynamic trafficking of FgVps17-GFP.

**Additional file 3: Video S2.** The time lapse of FgVps17-GFP+*ΔFgrab7* was monitored by laser confocal scanning microscopy.

**Additional file 4: Video S3.** The time lapse of FgVps17-GFP+*ΔFgrab5A* was monitored by laser confocal scanning microscopy.

**Additional file 5: Video S4.** The time lapse of FgVps17-GFP+*ΔFgrab5B* was monitored by laser confocal scanning microscopy.

**Additional file 6: Video S5.** The dynamic trafficking of FgVps35-GFP.

**Additional file 7: Video S6.** The time lapse of FgVps35-GFP+*ΔFgrab7* was monitored by laser confocal scanning microscopy.

**Additional file 8: Video S7.** The time lapse of FgVps35-GFP+*ΔFgrab5A* was monitored by laser confocal scanning microscopy.

**Additional file 9: Video S8.** The time lapse of FgVps35-GFP+*ΔFgrab5B* was monitored by laser confocal scanning microscopy.

## Data Availability

All the data supporting the findings contained in this manuscript (including supporting information) are provided in the submission and can be shared publicly after acceptance of the manuscript for publication by Stress Biology.
