# Supplementary material for: FgRab5 and FgRab7 are essential for endosomes biogenesis and non-redundantly recruit the retromer complex to the endosomes in Fusarium graminearum
Source: Stress Biol. 2021 Dec 6;1(1):17. doi: 10.1007/s44154-021-00020-3 (PMC10441910; doi:10.1007/s44154-021-00020-3)
Supplement: Supplementary file 1 — Additional file 1. [file 44154_2021_20_MOESM1_ESM.docx]

**Table S1: Fungal strains used in this study**

| Strain | Genotype description | Reference |
| --- | --- | --- |
| PH-1 | Wild-type |  |
| *ΔFgrab2* | deletion mutant from PH-1 | (Zheng et al., 2015) |
| *ΔFgrab4* | deletion mutant from PH-1 | (Zheng et al., 2015) |
| *ΔFgrab5A* | deletion mutant from PH-1 | (Zheng et al., 2015) |
| *ΔFgrab5B* | deletion mutant from PH-1 | (Zheng et al., 2015) |
| *ΔFgyptA* | deletion mutant from PH-1 | (Zheng et al., 2015) |
| *ΔFgrabX* | deletion mutant from PH-1 | (Zheng et al., 2015) |
| *ΔFgrab7* | deletion mutant from PH-1 | (Zheng et al., 2015) |
| *ΔFgrab8* | deletion mutant from PH-1 | (Zheng et al., 2015) |
| *PH-1+FgVps17-GFP* | PH-1 strain expressing FgVps17-GFP construct | This study |
| *ΔFgrab2+FgVps17-GFP* | *ΔFgrab2* strain expressing FgVps17-GFP construct | This study |
| *ΔFgrab4+FgVps17-GFP* | *ΔFgrab4* strain expressing FgVps17-GFP construct | This study |
| *ΔFgrab5A+FgVps17-GFP* | *ΔFgrab5A* strain expressing FgVps17-GFP construct | This study |
| *ΔFgrab5B+FgVps17-GFP* | *ΔFgrab5B* strain expressing FgVps17-GFP construct | This study |
| *ΔFgyptA+FgVps17-GFP* | *ΔFgyptA* strain expressing FgVps17-GFP construct | This study |
| *ΔFgrabX+FgVps17-GFP* | *ΔFgrabX* strain expressing FgVps17-GFP construct | This study |
| *ΔFgrab7+FgVps17-GFP* | *ΔFgrab7* strain expressing FgVps17-GFP construct | This study |
| *ΔFgrab8+FgVps17-GFP* | *ΔFgrab8* strain expressing FgVps17-GFP construct | This study |
| *PH-1+FgVps35-GFP* | PH-1 strain expressing FgVps35-GFP construct | This study |
| *ΔFgrab2+FgVps35-GFP* | *ΔFgrab2* strain expressing FgVps35-GFP construct | This study |
| *ΔFgrab4+FgVps35-GFP* | *ΔFgrab4* strain expressing FgVps35-GFP construct | This study |
| *ΔFgrab5A+FgVps35-GFP* | *ΔFgrab5A* strain expressing FgVps35-GFP construct | This study |
| *ΔFgrab5B+FgVps35-GFP* | *ΔFgrab5B* strain expressing FgVps35-GFP construct | This study |
| *ΔFgyptA+FgVps35-GFP* | *ΔFgyptA* strain expressing FgVps35-GFP construct | This study |
| *ΔFgrabX+FgVps35-GFP* | *ΔFgrabX* strain expressing FgVps35-GFP construct | This study |
| *ΔFgrab7+FgVps35-GFP* | *ΔFgrab7* strain expressing FgVps35-GFP construct | This study |
| *ΔFgrab8+FgVps35-GFP* | *ΔFgrab8* strain expressing FgVps35-GFP construct | This study |
| *PH-1+mCherry-FgRab7+FgVps35-GFP* | PH-1 strain expressing mCherry-FgRab7 and FgVps35-GFP constructs | This study |
| *PH-1+mCherry-FgRab7+FgVps17-GFP* | PH-1 strain expressing mCherry-FgRab7 and FgVps17-GFP constructs | This study |
| *PH-1+mCherry-FgRab5B+FgVps35-GFP* | PH-1 strain expressing mCherry-FgRab5B and FgVps35-GFP constructs | This study |
| *PH-1+mCherry-FgRab5B+FgVps17-GFP* | PH-1 strain expressing mCherry-FgRab5B and FgVps17-GFP constructs | This study |
| *PH-1+FgTom20-RFP* | PH-1 strain expressing FgTom20-RFP constructs | This study |
| *PH-1+FgTom20-RFP-FgRab7+FgVps17-GFP* | PH-1 strain expressing FgTom20-RFP-FgRab7 and FgVps17-GFP constructs | This study |
| *PH-1+FgTom20-RFP-FgRab7+FgVps35-GFP* | PH-1 strain expressing FgTom20-RFP-FgRab7 and FgVps35-GFP constructs | This study |
| *PH-1+FgTom20-RFP-FgRab5B+FgVps17-GFP* | PH-1 strain expressing FgTom20-RFP-FgRab5B and FgVps17-GFP constructs | This study |
| *PH-1+FgTom20-RFP-FgRab5B+FgVps35-GFP* | PH-1 strain expressing FgTom20-RFP-FgRab5B and FgVps35-GFP constructs | This study |
